# Coronary artery disease in atrial fibrillation ablation: impact on arrhythmic outcomes

**DOI:** 10.1093/europace/euad328

**Published:** 2023-12-08

**Authors:** Ida Anna Cappello, Luigi Pannone, Domenico Giovanni Della Rocca, Antonio Sorgente, Alvise Del Monte, Sahar Mouram, Giampaolo Vetta, Rani Kronenberger, Robbert Ramak, Ingrid Overeinder, Gezim Bala, Alexandre Almorad, Erwin Ströker, Juan Sieira, Mark La Meir, Dries Belsack, Andrea Sarkozy, Pedro Brugada, Kaoru Tanaka, Gian Battista Chierchia, Ali Gharaviri, Carlo de Asmundis

**Affiliations:** Heart Rhythm Management Centre, Postgraduate Program in Cardiac Electrophysiology and Pacing, Universitair Ziekenhuis Brussel—Vrije Universiteit Brussel, European Reference Networks Guard-Heart, Laarbeeklaan 101, 1090 Brussels, Belgium; Heart Rhythm Management Centre, Postgraduate Program in Cardiac Electrophysiology and Pacing, Universitair Ziekenhuis Brussel—Vrije Universiteit Brussel, European Reference Networks Guard-Heart, Laarbeeklaan 101, 1090 Brussels, Belgium; Heart Rhythm Management Centre, Postgraduate Program in Cardiac Electrophysiology and Pacing, Universitair Ziekenhuis Brussel—Vrije Universiteit Brussel, European Reference Networks Guard-Heart, Laarbeeklaan 101, 1090 Brussels, Belgium; Heart Rhythm Management Centre, Postgraduate Program in Cardiac Electrophysiology and Pacing, Universitair Ziekenhuis Brussel—Vrije Universiteit Brussel, European Reference Networks Guard-Heart, Laarbeeklaan 101, 1090 Brussels, Belgium; Heart Rhythm Management Centre, Postgraduate Program in Cardiac Electrophysiology and Pacing, Universitair Ziekenhuis Brussel—Vrije Universiteit Brussel, European Reference Networks Guard-Heart, Laarbeeklaan 101, 1090 Brussels, Belgium; Heart Rhythm Management Centre, Postgraduate Program in Cardiac Electrophysiology and Pacing, Universitair Ziekenhuis Brussel—Vrije Universiteit Brussel, European Reference Networks Guard-Heart, Laarbeeklaan 101, 1090 Brussels, Belgium; Heart Rhythm Management Centre, Postgraduate Program in Cardiac Electrophysiology and Pacing, Universitair Ziekenhuis Brussel—Vrije Universiteit Brussel, European Reference Networks Guard-Heart, Laarbeeklaan 101, 1090 Brussels, Belgium; Cardiac Surgery Department, Universitair Ziekenhuis Brussel—Vrije Universiteit Brussel, Brussels, Belgium; Heart Rhythm Management Centre, Postgraduate Program in Cardiac Electrophysiology and Pacing, Universitair Ziekenhuis Brussel—Vrije Universiteit Brussel, European Reference Networks Guard-Heart, Laarbeeklaan 101, 1090 Brussels, Belgium; Heart Rhythm Management Centre, Postgraduate Program in Cardiac Electrophysiology and Pacing, Universitair Ziekenhuis Brussel—Vrije Universiteit Brussel, European Reference Networks Guard-Heart, Laarbeeklaan 101, 1090 Brussels, Belgium; Heart Rhythm Management Centre, Postgraduate Program in Cardiac Electrophysiology and Pacing, Universitair Ziekenhuis Brussel—Vrije Universiteit Brussel, European Reference Networks Guard-Heart, Laarbeeklaan 101, 1090 Brussels, Belgium; Heart Rhythm Management Centre, Postgraduate Program in Cardiac Electrophysiology and Pacing, Universitair Ziekenhuis Brussel—Vrije Universiteit Brussel, European Reference Networks Guard-Heart, Laarbeeklaan 101, 1090 Brussels, Belgium; Heart Rhythm Management Centre, Postgraduate Program in Cardiac Electrophysiology and Pacing, Universitair Ziekenhuis Brussel—Vrije Universiteit Brussel, European Reference Networks Guard-Heart, Laarbeeklaan 101, 1090 Brussels, Belgium; Heart Rhythm Management Centre, Postgraduate Program in Cardiac Electrophysiology and Pacing, Universitair Ziekenhuis Brussel—Vrije Universiteit Brussel, European Reference Networks Guard-Heart, Laarbeeklaan 101, 1090 Brussels, Belgium; Cardiac Surgery Department, Universitair Ziekenhuis Brussel—Vrije Universiteit Brussel, Brussels, Belgium; Department of Radiology, Universitair Ziekenhuis Brussel—Vrije Universiteit Brussel, Brussels, Belgium; Heart Rhythm Management Centre, Postgraduate Program in Cardiac Electrophysiology and Pacing, Universitair Ziekenhuis Brussel—Vrije Universiteit Brussel, European Reference Networks Guard-Heart, Laarbeeklaan 101, 1090 Brussels, Belgium; Heart Rhythm Management Centre, Postgraduate Program in Cardiac Electrophysiology and Pacing, Universitair Ziekenhuis Brussel—Vrije Universiteit Brussel, European Reference Networks Guard-Heart, Laarbeeklaan 101, 1090 Brussels, Belgium; Department of Radiology, Universitair Ziekenhuis Brussel—Vrije Universiteit Brussel, Brussels, Belgium; Heart Rhythm Management Centre, Postgraduate Program in Cardiac Electrophysiology and Pacing, Universitair Ziekenhuis Brussel—Vrije Universiteit Brussel, European Reference Networks Guard-Heart, Laarbeeklaan 101, 1090 Brussels, Belgium; Heart Rhythm Management Centre, Postgraduate Program in Cardiac Electrophysiology and Pacing, Universitair Ziekenhuis Brussel—Vrije Universiteit Brussel, European Reference Networks Guard-Heart, Laarbeeklaan 101, 1090 Brussels, Belgium; Heart Rhythm Management Centre, Postgraduate Program in Cardiac Electrophysiology and Pacing, Universitair Ziekenhuis Brussel—Vrije Universiteit Brussel, European Reference Networks Guard-Heart, Laarbeeklaan 101, 1090 Brussels, Belgium

**Keywords:** Coronary artery disease, Atrial fibrillation, Cardiac computed tomography, Percutaneous coronary intervention, Coronary artery bypass graft

## Abstract

**Aims:**

Catheter ablation (CA) is an established treatment for atrial fibrillation (AF). A computed tomography (CT) may be performed before ablation to evaluate the anatomy of pulmonary veins. The aim of this study is to investigate the prevalence of patients with coronary artery disease (CAD) detected by cardiac CT scan pre-ablation and to evaluate the impact of CAD and revascularization on outcomes after AF ablation.

**Methods and results:**

All consecutive patients with AF diagnosis, hospitalized at Universitair Ziekenhuis Brussel, Belgium, between 2015 and 2019, were prospectively screened for enrolment in the study. Inclusion criteria were (i) AF diagnosis, (ii) first procedure of AF ablation with cryoballoon CA, and (iii) contrast CT scan performed pre-ablation. A total of 576 consecutive patients were prospectively included and analysed in this study. At CT scan, 122 patients (21.2%) were diagnosed with CAD, of whom 41 patients (7.1%) with critical CAD. At survival analysis, critical CAD at CT scan was a predictor of atrial tachyarrhythmia (AT) recurrence during the follow-up, only in Cox univariate analysis [hazard ratio (HR) = 1.79] but was not an independent predictor in Cox multivariate analysis. At Cox multivariate analysis, independent predictors of AT recurrence were as follows: persistent AF (HR = 2.93) and left atrium volume index (HR = 1.04).

**Conclusion:**

In patients undergoing CT scan before AF ablation, critical CAD was diagnosed in 7.1% of patients. Coronary artery disease and revascularization were not independent predictors of recurrence; thus, in this patient population, AF ablation should not be denied and can be performed together with CAD treatment.

## Graphical Abstract

**Graphical Abstract:**
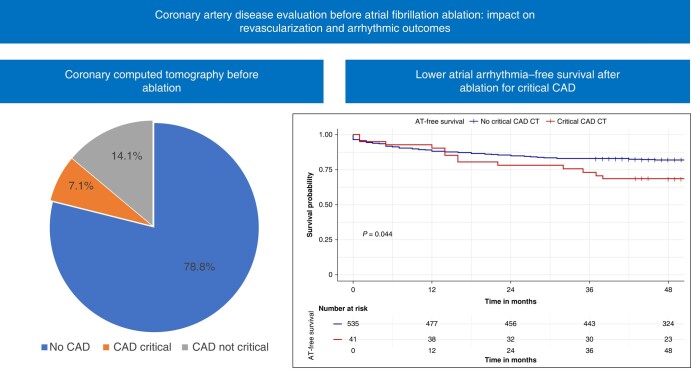
In patients undergoing computed tomography (CT) scan before atrial fibrillation ablation, critical coronary artery disease (CAD) was diagnosed in 7.1% of patients. Patients without critical CAD at CT scan (Group 1) had higher atrial tachyarrhythmia–free survival during the follow-up, compared with patients found with critical CAD at CT scan (Group 2). However, CAD was not an independent predictor of recurrence after ablation.

**What’s new?**

- Coronary artery disease (CAD) is diagnosed in 21.2% of patients, and critical CAD is diagnosed in 7% of patients with a coronary computed tomography (CT) scan before atrial fibrillation (AF) ablation.
- Coronary artery disease and revascularization were not independent predictors of atrial tachyarrhythmia (AT) recurrence during the follow-up in Cox multivariate analysis. At Cox multivariate analysis, independent predictors of AT recurrence were as follows: persistent AF [hazard ratio (HR) = 2.93] and left atrium volume index (HR = 1.04). Thus, in this patient population, AF catheter ablation appears to be as effective as in patients without CAD/revascularization.

## Introduction

Atrial fibrillation (AF) is one of the most frequent cardiac arrhythmias. Its prevalence has increased over the last 20 years, and it has been estimated that will continue to increase over the next 30 years. In 2050, estimated 17.9 million people will suffer this condition in Europe.^1^ Nowadays, catheter ablation (CA) using radiofrequency or cryoenergy is an established treatment for AF.^2,3^ Before the ablation treatment, a computed tomography (CT) scanner to evaluate the anatomy of pulmonary veins (PVs) and for procedural planning may be performed.^4–6^

Recent studies have demonstrated that 256-slice cardiac CT scanners have the ability to assess the entire coronary tree demonstrating to have high diagnostic accuracy for the identification of anatomically significant coronary artery disease (CAD), generally defined as coronary artery stenoses with a lumen diameter reduction of at least 50%.^7,8^ However, the anatomically significant appearance of a coronary stenosis does not always equate with functional significance, and this is particularly true for intermediate-type coronary lesions.^9,10^

Computed tomography scan has been demonstrated feasible in patients with AF.^11,12^ The diagnostic yield of coronary CT for CAD in AF patients is up to 20.0–39.0%,^11,13^ confirmed at invasive cardiac catheterization in ≈10.0–15.0%.^11,14^ This might lead to revascularization in ≈7.0–10.0% of patients.^11,14^

However, in the current guidelines, there are no recommendations about the use of the CT scan in the contest of screening of pre-AF ablation.^2,15^

Thus, the aim of this study is (i) to assess the role of routine cardiac CT scan before AF ablation in evaluating coronary artery lesions in patients with AF, (ii) to investigate the prevalence of patients with critical CAD (>70%) detected by cardiac CT scan pre-ablation and confirmed by invasive coronary angiography, and (iii) to evaluate the impact of critical CAD and revascularization on the clinical outcomes after AF ablation.

## Methods

### Study population

All consecutive patients with AF diagnosis, hospitalized at Universitair Ziekenhuis Brussel, Belgium, between March 2015 and October 2019, were prospectively screened for enrolment in the study and followed up.

Inclusion criteria were (i) AF diagnosis following current guidelines,^2^ (ii) first procedure of AF ablation with cryoballoon CA, and (iii) contrast CT scan performed pre-ablation.

Exclusion criteria were as follows: (i) previous left-side ablation and (ii) contraindication to contrast CT scan, including chronic kidney dysfunction.^16^ A total of 1537 patients were screened and 576 with a CT scan available were enrolled.

The following data were collected on electronic medical record: (i) demographic data, (ii) previous history of heart failure, (iii) previous critical CAD (stenosis more than 70%) revascularized with percutaneous coronary intervention (PCI) or with coronary artery bypass graft (CABG), and (iv) echocardiographic data including left ventricular ejection fraction (LVEF) and left atrium volume index (LAVi). Data were carefully reviewed and confirmed by two independent researchers (I.A.C. and L.P.), both blinded to cardiac arrhythmia occurrence, to guarantee the accuracy of the data extraction.

### Computed tomography scan analysis

**Figure 1:**
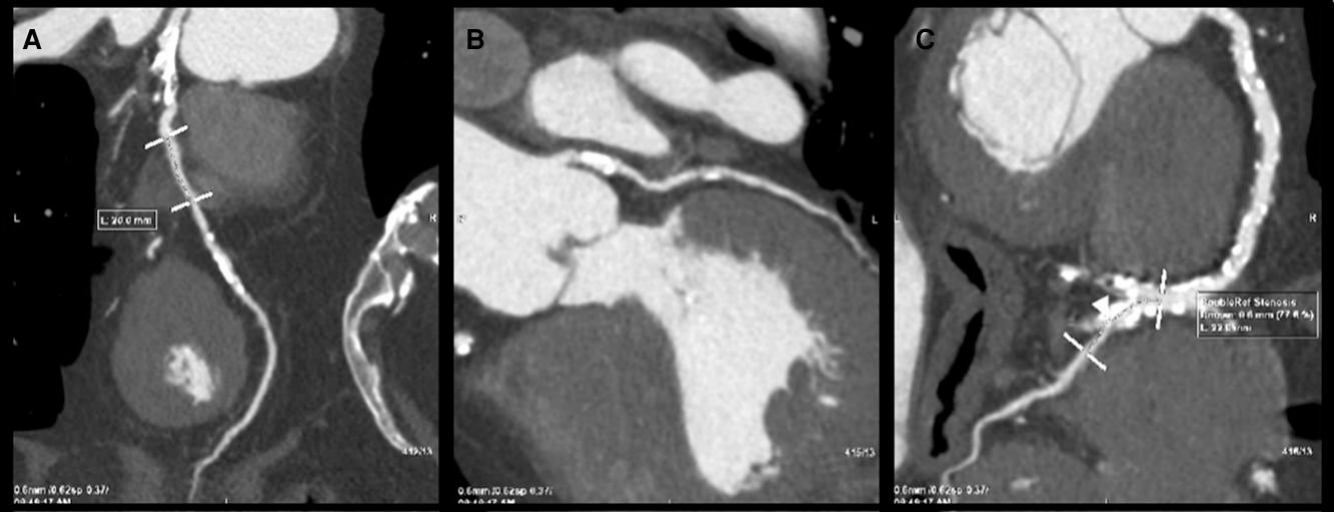
Computed tomography of critical coronary artery disease at pre-ablation scan. (*A*) Curved multiplanar (MPR) image of severe stenosis in proximal left anterior descending (LAD) caused by a calcified plaque. (*B*) Curved MPR image of severe stenosis in the proximal LCX caused by a calcified plaque. (*C*) Curved MPR image of a severe stenosis in the proximal RCA caused by both calcified and non-calcified plaques.

At admission, all patients underwent to a CT 256-Slice Scanner (GE Healthcare system). All cardiac CT scans were assessed at heart window using 2D axial, coronal, and sagittal planes, following current guidelines.^16,17^ Briefly, a heart rate of <60 bpm was targeted. Special attention was paid to proper positioning of patient and electrocardiogram (ECG) leads for prospectively ECG-triggered axial acquisition. The scan range was typically from below the tracheal bifurcation or the mid-level of the left pulmonary artery to just below the lower cardiac border. Iodine contrast was administered, aiming at high intra-arterial opacification of more than 250 Hounsfield units. Two experienced radiologists, both blinded to clinical follow-up, performed the CT image analysis. Computed tomography scan segmentation was performed with (GE AW Server) by two independent experienced radiologists (K.T. and D.B.), and the stenosis was quantified for the following vessels: left anterior descending (LAD), left circumflex (LCx), and right coronary artery (RCA). The stenosis was defined as critical if >70% following current guidelines^18^ (*Figure 1*). Based on this definition at CT scan, patients were divided in two groups, namely: Group 1 including both patients with no CAD at all and with no critical CAD at CT scan and Group 2 with critical CAD at CT scan.

Patients with a critical stenosis at CT scan pre-ablation underwent a coronary angiography and eventually revascularization, if deemed indicated following current guidelines.^18^ Critical CAD at coronary angiography was defined visually or by fractional flow reserve based on current guidelines.^18^

### Ablation procedure

All patients underwent pulmonary vein isolation (PVI) with Arctic Front Advance PRO™ CB-A system. Our standard pre-procedural management and ablation for CB-A has been previously described in detail.^19–22^

All procedures were performed under general anaesthesia and under oesophageal temperature monitoring. Cryoenergy applications were interrupted in case of luminal oesophageal temperatures (LET) < 15°C. After having obtained left atrium (LA) access, through a steerable sheath (FlexCath Advance Medtronic Inc. 15Fr), a 28 mm CB-A catheter (Arctic Front Advance PRO, Medtronic Inc.) was advanced in the LA for PVI and an inner lumen mapping catheter (Achieve, Medtronic Inc.) was positioned in each PV ostium. The CB-A was advanced, inflated, and positioned at each PV ostium. Optimal vessel occlusion was defined by selective contrast injection. Once vessel occlusion was deemed satisfactory, delivery of cryoenergy to allow freezing was commenced. Standard cryothermal applications lasted 180 s. Our target temperature was −40°C within the first 60 s. If the temperature did not attain this value, an extra freeze was delivered. The ablation sequence was the following: left superior PV (LSPV), left inferior PV (LIPV), right inferior PV (RIPV), and right superior PV (RSPV). In order to avoid phrenic nerve palsy (PNP), diaphragmatic stimulation was achieved by pacing the phrenic nerve during septal PV ablation.

### Post-procedural management and follow-up

All patients underwent continuous telemetry monitoring for at least 24 h after the procedure and were discharged after overnight observation if no complications occurred. Before discharge, transthoracic echocardiography and venous Doppler ultrasound were performed in all patients. Oral anticoagulation was started the same evening of ablation and continued for at least 2 months after the procedure and then according to patient’s thromboembolic risk profile. Antiarrhythmic drugs (AADs) were continued for 2 months after ablation and then according to clinical judgement and patient preference.

Our institutional clinical follow-up strategy included in-person outpatient visits at 1, 3, 6, and 12 months after ablation for the first year and then every 6 months. At each visit, clinical examination and a 12-lead ECG were performed. Furthermore, a 7-day Holter monitoring was recorded at 3, 6, and 12 months for the first year and then every 12 months.

The primary safety endpoint included any major peri-procedural complications (e.g. death, stroke, pericardial effusion/tamponade with/without surgical treatment, myocardial infarction, phrenic nerve paralysis, and serious vascular complications) occurring within 7 days post-procedure. Serious vascular complications were defined as the following: pseudoaneurysm, arteriovenous fistula, and retroperitoneal bleeding and haematoma/bleeding requiring surgery or blood transfusion. Atrio-oesophageal fistula occurrence at long-term was also evaluated.

The primary endpoint was arrhythmia-free survival during follow-up. Arrhythmia recurrence was defined as any documented (at ECG or Holter) atrial tachyarrhythmias (AT) lasting more than 30 s after a 90-day post-ablation blanking period. The secondary endpoint was critical CAD diagnosed at CT scan.

### Statistical analysis

All variables were tested for normality with Shapiro–Wilk test. Normally distributed variables were described as mean ± standard deviation, and the groups were compared through ANOVA and paired or unpaired *t*-test as appropriate, while the non-normally distributed variables were described as median (interquartile range) and compared by Mann–Whitney test or Wilcoxon signed-rank test as appropriate. The categorical variables were described as frequencies (percentages) and compared by χ^2^ test or Fisher’s exact test as appropriate.

Kaplan–Meier’s curves were drawn to describe the patients’ freedom from arrhythmias during the follow-up period and log-rank test was used.

Cox’s proportional hazard model was performed to identify risk factors for AF recurrence. The covariates entered in the univariate and multivariate Cox model were chosen according to their clinical significance. Variables with *P* < 0.10 were then entered in the multivariate model and selected with a backward stepwise approach. The proportional hazards assumption for the Cox model was tested with cox.zph function. The minimum number of events per predictor variable was set at 5 as recommended to avoid overfitting problem.^23,24^

A *P* value <0.05 was considered statistically significant.

The analysis was performed using R software version 3.6.2 (R Foundation for Statistical Computing, Vienna, Austria).

## Results

### Study population characteristics

Five-hundred and seventy-six consecutive patients were prospectively included and analysed in this study. At CT scan performed before AF ablation, 122 patients (21.2%) were diagnosed with CAD, of whom 41 patients (7.1%) with critical CAD (*Figure 1*). Patients with critical CAD at CT scan were included in Group 2 (*n* = 41) and all other patients were included in Group 1 (*n* = 535).

Compared with patients without critical CAD at CT scan (Group 1), patients with critical CAD at CT scan (Group 2) showed higher mean age at procedure (69.1 years ± 9.6 vs. 60.8 years ± 12.6, *P* < 0.001), lower prevalence of paroxysmal AF [17 patients (41.5%) vs. 334 patients (62.4%), *P* = 0.032], higher prevalence of heart failure history [5 patients (12.2%) vs. 20 patients (3.7%), *P* = 0.005], and higher CHA_2_DS_2_VASc score (2.2 ± 1.5 vs. 1.6 ± 1.3, *P* = 0.026). There was no difference in drug treatment between the two groups except for Class III AADs [17 patients (41.5%) vs. 124 patients (23.2%), *P* = 0.001].

Complete patient characteristics are summarized in *Table 1* and Supplementary material online, *Table S1*.

**Table 1:** Clinical characteristics of patients with and without critical coronary artery disease

	Group 1	Group 2	Total	*P* value
	(*n* = 535)	(*n* = 41)	(*n* = 576)	
Age at ablation (years)	60.8 ± 12.6	69.1 ± 9.6	61.4 ± 12.6	<0.001
Male (*n*, %)	319 (59.6%)	27 (65.9%)	346 (60.1%)	0.43
BMI (kg/m^2^)	28.0 ± 11.5	28.5 ± 4.8	28.1 ± 11.2	0.84
AF paroxysmal (*n*, %)	334 (62.4%)	17 (41.5%)	351 (60.9%)	0.032
Hypertension (*n*, %)	205 (38.3%)	17 (41.5%)	222 (38.5%)	0.24
Diabetes (*n*, %)	46 (8.6%)	4 (9.8%)	50 (8.7%)	0.65
Dyslipidaemia (*n*, %)	130 (24.3%)	14 (34.1%)	144 (25.0%)	0.06
Heart failure history (*n*, %)	20 (3.7%)	5 (12.2%)	25 (4.3%)	0.005
Chronic kidney disease (*n*, %)	25 (4.7%)	4 (9.8%)	29 (5.0%)	0.12
Creatininemia (mg/dL)	0.8 ± 0.4	0.9 ± 0.4	0.8 ± 0.4	0.13
GFR MDRD (mL/min)	78.5 ± 18.6	76.7 ± 22.2	78.3 ± 18.8	0.65
TIA or stroke (*n*, %)	21 (3.9%)	0 (0.0%)	21 (3.6%)	0.22
CHA_2_DS_2_VASc score	1.6 ± 1.3	2.2 ± 1.5	1.6 ± 1.4	0.026
LVEF (%)	56.1 ± 9.8	56.8 ± 10.6	56.2 ± 9.9	0.78
LAVi (mL/m^2^)	36.0 ± 11.6	42.8 ± 11.3	36.5 ± 11.6	0.09
Drugs				
Class Ic (*n*, %)	107 (20.0%)	5 (12.2%)	112 (19.4%)	0.31
Class II (*n*, %)	147 (27.5%)	11 (26.8%)	158 (27.4%)	0.77
Class III (*n*, %)	124 (23.2%)	17 (41.5%)	141 (24.5%)	0.001
Class IV (*n*, %)	9 (1.7%)	2 (4.9%)	11 (1.9%)	0.11
DOAC (*n*, %)	293 (54.8%)	24 (58.5%)	317 (55.0%)	0.17
Aspirin (*n*, %)	35 (6.5%)	3 (7.3%)	38 (6.6%)	0.72

Critical and no critical CAD refers to CT scan.

AF, atrial fibrillation; BMI, body mass index; CAD, coronary artery disease; CT, computed tomography scan; DOAC, direct oral anticoagulants; GFR, glomerular filtration rate; LAVi, left atrium volume index; LVEF, left ventricular ejection fraction; MDRD, Modification of Diet in Renal Disease; TIA, transient ischaemic attack.

### Computed tomography, coronary angiography results, and revascularization

Among patients with critical CAD at CT scan (Group 2), the affected vessels were as follows: LAD in 29 patients (70.7%), LCx in 11 patients (26.8%), and RCA in 14 patients (34.1%) (*Table 2* ). Of these 41 patients, 39 patients (95.1%) were first diagnosed with CAD after CT scan pre-ablation.

**Table 2:** Computed tomography and coronary angiography characteristics of patients with and without critical coronary artery disease

	Group 1	Group 2	Total	*P* value
	(*n* = 535)	(*n* = 41)	(*n* = 576)	
CT CAD (*n*, %)	81 (15.1%)	41 (100.0%)	122 (21.2%)	<0.001
CT CAD not critical (*n*, %)	80 (15.0%)	18 (43.9%)	98 (17.0%)	<0.001
CT CAD not critical LAD (*n*, %)	73 (13.6%)	5 (12.2%)	78 (13.5%)	0.79
CT CAD not critical LCx (*n*, %)	19 (3.6%)	7 (17.1%)	26 (4.5%)	<0.001
CT CAD not critical RCA (*n*, %)	28 (5.2%)	10 (24.4%)	38 (6.6%)	<0.001
CT CAD critical LAD (*n*, %)	0 (0.0%)	29 (70.7%)	29 (5.0%)	<0.001
CT CAD critical LCx (*n*, %)	0 (0.0%)	11 (26.8%)	11 (1.9%)	<0.001
CT CAD critical RCA (*n*, %)	0 (0.0%)	14 (34.1%)	14 (2.4%)	<0.001
Coronary angiography (*n*, %)	47 (45.6%)	31 (75.6%)	78 (54.2%)	0.001
Coronary angiography CAD (*n*, %)	37 (6.9%)	30 (73.2%)	67 (11.6%)	<0.001
Coronary angiography CAD not critical (*n*, %)	31 (5.9%)	23 (57.5%)	54 (9.5%)	<0.001
Coronary angiography CAD not critical LAD (*n*, %)	23 (4.4%)	14 (35.0%)	37 (6.5%)	<0.001
Coronary angiography CAD not critical LCx (*n*, %)	9 (1.7%)	10 (25.0%)	19 (3.4%)	<0.001
Coronary angiography CAD not critical RCA (*n*, %)	10 (1.9%)	10 (25.0%)	20 (3.5%)	<0.001
Coronary angiography CAD critical (*n*, %)	14 (2.7%)	19 (47.5%)	33 (5.8%)	<0.001
Coronary angiography CAD critical LAD (*n*, %)	11 (2.1%)	10 (25.6%)	21 (3.8%)	<0.001
Coronary angiography CAD critical LCx (*n*, %)	4 (0.8%)	7 (17.9%)	11 (2.0%)	<0.001
Coronary angiography CAD critical RCA (*n*, %)	5 (1.0%)	7 (17.9%)	12 (2.2%)	<0.001

Critical and no critical CAD refers to CT scan.

CAD, coronary artery disease; CT, computed tomography; LAD, left anterior descending; LCx, left circumflex; RCA, right coronary artery.

Coronary angiography was performed in all patients with critical CAD at CT scan after a median of 2 days (0–3) from the CT. A critical stenosis in at least one coronary artery was confirmed at coronary angiography in 19 patients (47.5% of patients with a critical stenosis at CT scan). A critical stenosis was found at coronary angiography as follows: LAD in 10 patients (25.6%), LCx in 7 patients (17.9%), and RCA in 7 patients (17.9%), (*Table 2*). Out of 19 patients with critical CAD at angiography, 14 patients (73.7%) were revascularized with PCI or with CABG. In five patients (26.3%), a conservative medical therapy was chosen after heart team discussion. Revascularization procedures occurred at a median of 3 days (0–5) from CT scan. In these patients, AF ablation was postponed and performed at a median of 73.0 days (39.3–90.3) after revascularization.

Out of 576 patients, 556 patients had no prior CAD documented, so no critical CAD or status of CAD prior to CT scan unknown [517 patients (96.6%) in Group 1 vs. 39 patients (95.1%) in Group 2, *P* = 0.61].

Twenty patients had a previous revascularization with PCI or CABG at a median 33 months (5.0–54.0) before AF ablation. One patient had a revascularization both before and after ablation. All revascularizations were deemed as complete at the end of the revascularization procedure with no complications observed.

### Follow-up and predictors of the primary endpoint

After a median follow-up of 27.3 months (16.4–32.2), 109 patients (18.9%) experienced AT recurrence after AF ablation. Atrial tachyarrhythmia recurrence occurred at a median follow-up of 15.8 months (6.2–19.4).

Two patients (0.3%) died during the follow-up for non-cardiological cause. There were no peri-procedural deaths. The primary safety endpoint occurred in nine patients (1.5%) with nine phrenic nerve paralysis. No atrio-oesophageal fistula occurred.

**Figure 2:**
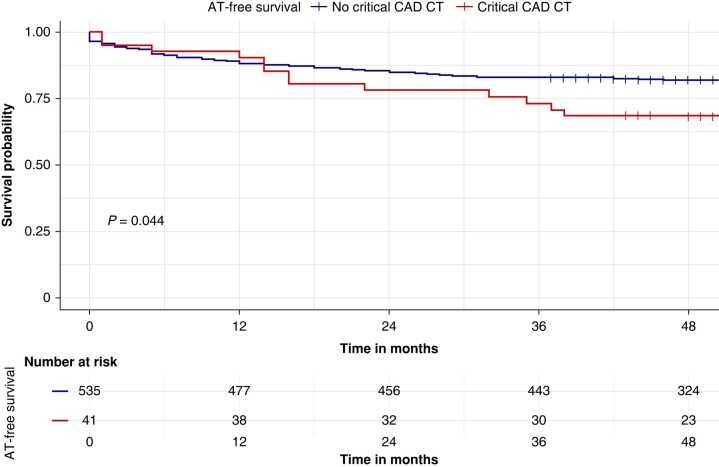
Kaplan–Meier curves of survival free from atrial tachyarrhythmia (AT) recurrence by critical coronary artery disease at CT scan. Kaplan–Meier curve of survival free from AT recurrence by critical coronary artery disease (CAD) at CT scan; patients in Group 1 (blue curve) had higher AT-free survival during the follow-up compared with Group 2 (red curve) (92.7 vs. 78.1%, log-rank *P* = 0.002).

At survival analysis after AF ablation procedure, patients without critical CAD at CT scan (Group 1) had higher AT-free survival during the follow-up, compared with patients found with critical CAD at CT scan (Group 2) (82.1 vs. 68.3%, log-rank *P* = 0.004) (*Figure 2*).

**Figure 3:**
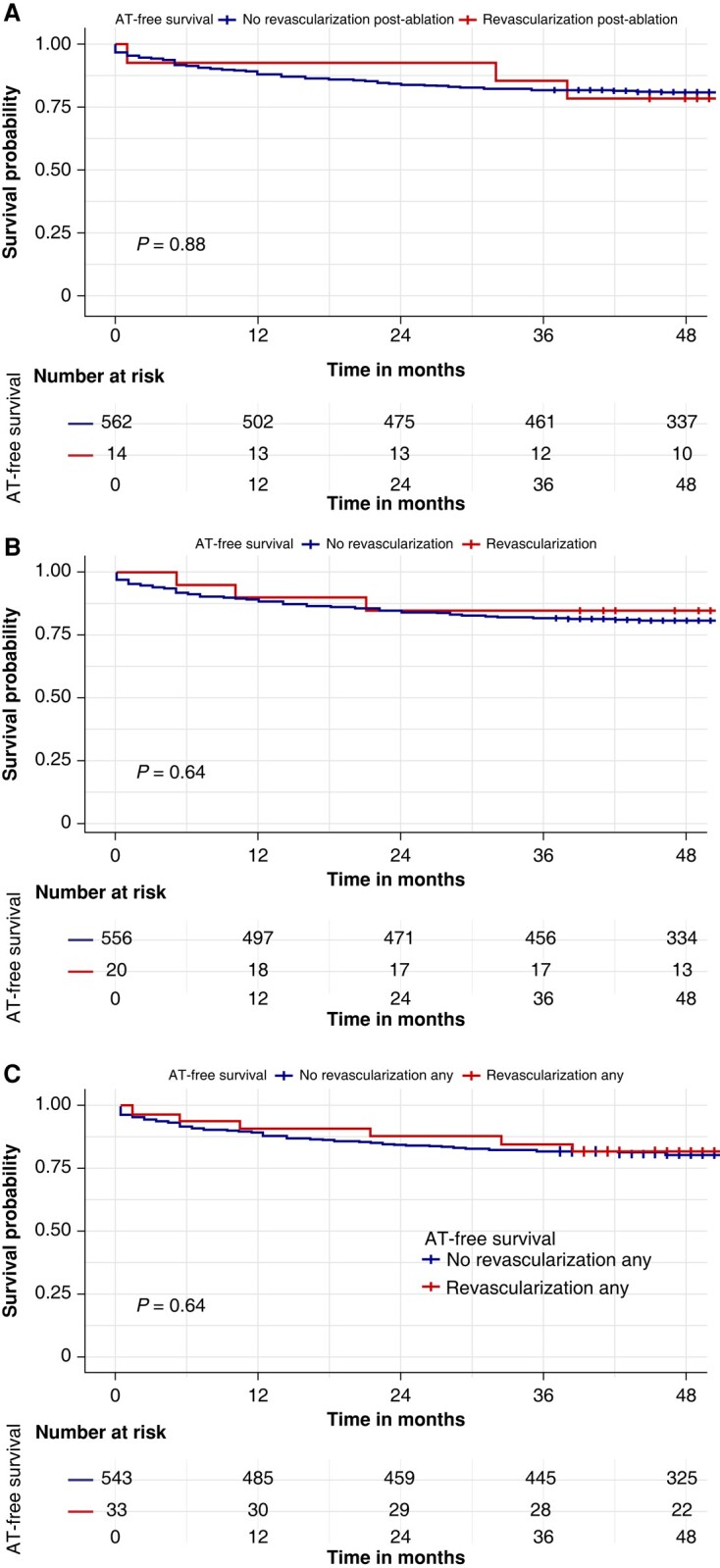
Kaplan–Meier curves of survival free from atrial tachyarrhythmia (AT) recurrence by revascularization. (*A*) Kaplan–Meier curve of survival free from AT recurrence by revascularization after atrial fibrillation (AF) ablation; there was no difference in AT-free survival between patients without (blue curve) and with (red curve) revascularization after AF ablation (78.6 vs. 81.1%, log-rank *P* = 0.88). (*B*) Kaplan–Meier curve of survival free from AT recurrence by revascularization history before AF ablation; there was no difference in AT-free survival between patients without (blue curve) and with (red curve) revascularization history before AF ablation (85.0 vs. 80.9%, log-rank *P* = 0.64). (*C*) Kaplan–Meier curve of survival free from AT recurrence by any revascularization; there was no difference in AT-free survival between patients without (blue curve) and with (red curve) any revascularization (81.8 vs. 81.0%, log-rank *P* = 0.86).

No significant difference in AT-free survival between patients with and without revascularization after ablation was found (78.6 vs. 81.1%, log-rank *P* = 0.88) (*Figure 3*). No significant difference in AT-free survival was found also between patients with and without a previous revascularization (85.0 vs. 80.9%, log-rank *P* = 0.64) (*Figure 3*). Finally, there was no difference in AT-free survival in the merged cohort of patients with any revascularization vs. patients with no previous revascularization (81.8 vs. 81.0%, log-rank *P* = 0.86) (*Figure 3*).

At Cox univariate analysis, predictors of AT recurrence were the following: age at ablation [hazard ratio (HR) = 1.03, confidence interval (CI) 95% 1.01–1.04, *P* = 0.002], persistent AF (HR = 2.22, CI 95% 1.43–3.44, *P* < 0.001), index LAVi (HR = 1.04, CI 95% 1.01–1.07, *P* = 0.009), and critical CAD at CT scan (HR = 1.79, CI 95% 1.01–3.2, *P* = 0.04) (*Table 3*).

**Table 3:** Cox univariate and multivariate analysis for atrial tachyarrhythmia recurrence

	Cox univariate analysis	Cox multivariate analysis
	HR (CI 95%), *P* value	HR (CI 95%), *P* value
Age at ablation (years)	1.03 (1.01–1.04), *P* = 0.002	
Gender (male)	0.96 (0.66–1.4), *P* = 0.85	
AF persistent	2.22 (1.43–3.44), *P* < 0.001	2.93 (1.29–6.65), *P* = 0.001
Hypertension	1.61 (0.85–2.51), *P* = 0.37	
Diabetes	1.23 (0.63–2.38), *P* = 0.55	
Index LAVi (mL/m^2^)	1.05 (1.02–1.08), *P* = 0.003	1.04 (1.01–1.07), *P* = 0.009
CAD critical at CT scan	1.79 (1.01–3.2), *P* = 0.04	
Revascularization (pre- or post-CT scan)	0.93 (0.41–2.1), *P* = 0.86	

AF, atrial fibrillation; CAD, coronary artery disease; CT, computed tomography; LAVi, left atrium volume index.

At Cox multivariate analysis, independent predictors of AT recurrence were as follows: persistent AF (HR = 2.93, CI 95% 1.29–6.65, *P* = 0.001) and index LAVi (HR = 1.04, CI 95% 1.01–1.07, *P* = 0.009) (*Table 3*).

## Discussion

The main findings of this study can be summarized as follows: (i) CAD was diagnosed in 21.2% of patients, and a critical CAD was diagnosed in 7.1% of patients with a coronary CT scan before AF ablation; (ii) at survival analysis, patients without critical CAD at CT scan had higher AT-free survival during the follow-up, compared with patients that were found with critical CAD at CT scan. No significant difference in AT-free survival between patients with and without revascularization after ablation was found. (iii) At Cox multivariate analysis, independent predictors of AT recurrence were persistent AF and index LAVi.

### The role of computed tomography scan in detecting coronary artery disease in patients undergoing atrial fibrillation ablation

Computed tomography scan is an emerging technology to assess CAD and in the context of pre-procedural screening.

Different studies such as the SCOT-HEART validated the use of CT scan in the context of CAD.^25^ The combination of the strongest negative predictive value of CT, compared with the gold standard and a comparable positive predictive value compared with other modalities, in conjunction with being the least expensive investigation, demonstrated that CT was the most cost-effective first-line screening test at all disease prevalence (25, 45, and 75%).^26^

Coronary CT scan, in the context of AF ablation, is an appealing modality for left atrial appendage thrombus evaluation,^27^ PV anatomy analysis,^28^ and to rule out CAD.^29^ Following current guidelines,^18^ in high-risk asymptomatic adults, coronary CT may be considered for cardiovascular risk assessment and CAD evaluation. The cardiovascular risk of adult patients with AF is not low, as CAD prevalence in this population is up to 18–46.5%.^30^ In high-risk individuals, current guidelines recommend functional imaging or coronary CT scan.^31^ In our study, a critical CAD was diagnosed in 7.1% of patients with a coronary CT scan before AF ablation. However, 20 patients had previously known CAD for a total prevalence of CAD of 10.6% in the whole cohort, excluding non-critical CAD. Using a cut-off of 50% for critical CAD at CT scan, den Uijl et al.^32^ found a significant disease in 23% of patients undergoing AF ablation. This lower cut-off may explain the higher prevalence of CAD in the previous study.^32^

Our data are consistent with a previous study from Kralev et al.,^30^ demonstrating a stable CAD in 13% of patients with AF.

Computed tomography scan is a non-invasive technique that can identify at-risk patients in whom a coronary angiography is deemed indicated. Despite angiography confirming a critical stenosis at CT scan in ≈50% of patients, diagnosing CAD (critical or not) may have a prognostic impact. Indeed, in the SCOT-HEART, the use of CT resulted in a significantly lower rate of death from coronary heart disease or non-fatal myocardial infarction, without a significantly higher rate of coronary angiography or coronary revascularization. This effect is thought to be secondary to optimized medical therapy in the newly diagnosed CAD population.^25^ Indeed, at subgroup analysis, higher benefit was observed in the group with higher 10-year cardiovascular risk, according to the ASSIGN score.

### The role of ischaemic heart disease in the prognosis after atrial fibrillation ablation

The role of CAD in the prognosis of AF after ablation is a matter of debate. Indeed, CAD has been associated with AF^33^; however, the prevalence of AF among patients with proven CAD is low, at 0.2–5%.^34^ Previous studies in patients after AF ablation with radiofrequency demonstrated that the presence and extent of CAD did not impact the outcomes after AF CA with radiofrequency.^32,35^ The current study is the first to investigate the role of CAD and, in particular, of CAD revascularization on the prognosis after AF CA with cryoballoon CA. Our data demonstrated that patients with critical CAD show a lower survival free from AT, driven by higher arrhythmia recurrence. However, CAD was a predictor of recurrence at univariate but not at multivariate analysis. Thus, the presence of CAD itself cannot be considered as an independent risk factor; however, it may be a marker associated with a more aggressive clinical phenotype. Consistent with these findings, no significant difference in AT-free survival between patients with and without revascularization after ablation was found. Most likely, in patients without symptomatic CAD, other pathophysiological factors such as age and LAVi may contribute to AF initiation and perpetuation. Thus, in this patient population, AF CA should not be denied and can be performed in parallel with CAD treatment. Previous studies on radiofrequency AF CA focused on the presence and extent of CAD.^32,35^ The current study also investigated the role of revascularization on arrhythmic outcomes. Revascularization (pre- or post-CT scan) was not a predictor of AF recurrence. Thus, pre-ablation coronary angiography and, eventually, revascularization should be performed, if indicated by current guidelines.^18^ Indeed, revascularization has a demonstrated prognostic benefit in chronic CAD for proximal LAD disease (10 patients in our study).^31^ Pre-ablation coronary angiography and revascularization were associated with no complications. Furthermore, all complications after AF ablation were related to cryoablation (nine phrenic nerve paralysis). Thus, CAD invasive diagnosis and treatment may not prevent from AF ablation. However, postponing AF ablation after revascularization should be recommended. In the current study, AF ablation was performed at a median of 73.0 days after revascularization.

## Limitations

The current study is a retrospective single-centre study from a high-volume centre experienced with AF ablation. Radiofrequency or pulsed-field AF CA has not been investigated in the current study. The decision for revascularization was based on physician clinical judgement and current guidelines.^18^ Patients with chronic kidney dysfunction were excluded for higher risk of contrast-induced nephropathy. These results may not be extended to this patient cohort.

## Conclusions

In patients undergoing CT scan before AF ablation, critical CAD was diagnosed in 7.1% of patients. At survival analysis, patients without critical CAD by CT scan had higher AT-free survival during the follow-up, compared with patients found with critical CAD by CT scan. No significant difference in AT-free survival between patients with and without revascularization after ablation was found. At Cox multivariate analysis, independent predictors of AT recurrence were as follows: persistent AF and index LAVi.

## Supplementary Material

euad328_Supplementary_Data

## Data Availability

All relevant data are within the manuscript and its online Supplementary material.
