# Supplementary material for: Coronary artery disease in atrial fibrillation ablation: impact on arrhythmic outcomes
Source: Europace. 2023 Dec 8;25(12):euad328. doi: 10.1093/europace/euad328 (PMC10751806; doi:10.1093/europace/euad328)
Supplement: euad328_Supplementary_Data [file euad328_supplementary_data.docx]

# Supplemental material

# Coronary artery disease in atrial fibrillation ablation: impact on arrhythmic outcomes

# Supplemental Tables

## Supplemental Table 1. Clinical characteristics of patients with and without critical/non-critical coronary artery disease.

|  | **No CAD at CT**  **(N=535)** | **Non-Critical CAD at CT**  **(N=81)** | **Critical CAD at CT**  **(N=41)** | **Total**  **(N=576)** | **p value** |
| --- | --- | --- | --- | --- | --- |
| **Age at ablation (years)** | 59.9 ± 13.0 | 65.9 ± 8.7 | 69.1 ± 9.6 | 61.4 ± 12.6 | < 0.001 |
| **Male (n, %)** | 256 (47.8%) | 63 (77.8%) | 27 (65.9%) | 346 (60.1%) | 0.001 |
| **BMI (kg/m^2^)** | 28.1 ± 12.5 | 27.8 ± 4.8 | 28.5 ± 4.8 | 28.1 ± 11.2 | 0.95 |
| **AF paroxysmal (n, %)** | 281 (52.5%) | 53 (65.4%) | 17 (41.5%) | 351 (60.9%) | 0.082 |
| **Hypertension (n, %)** | 164 (30.7%) | 41 (50.6%) | 17 (41.5%) | 222 (38.5%) | 0.24 |
| **Diabetes (n, %)** | 37 (6.9%) | 9 (11.1%) | 4 (9.8%) | 50 (8.7%) | 0.81 |
| **Dyslipemia (n, %)** | 97 (18.1%) | 33 (40.7%) | 14 (34.1%) | 144 (25.0%) | 0.002 |
| **Heart failure history (n, %)** | 18 (3.4%) | 2 (2.5%) | 5 (12.2%) | 25 (4.3%) | 0.014 |
| **Chronic Kidney disease (n, %)** | 20 (3.7%) | 5 (6.2%) | 4 (9.8%) | 29 (5.0%) | 0.28 |
| **Creatininemia (mg/dl)** | 0.8 ± 0.3 | 0.8 ± 0.4 | 0.9 ± 0.4 | 0.8 ± 0.4 | 0.20 |
| **GFR MDRD (ml/min)** | 79.3 ± 19.2 | 74.6 ± 14.8 | 76.7 ± 22.2 | 78.3 ± 18.8 | 0.65 |
| **TIA or Stroke (n, %)** | 18 (3.4%) | 3 (3.7%) | 0 (0.0%) | 21 (3.6%) | 0.43 |
| **CHA_2_DS_2_VASc score** | 1.6 ± 1.4 | 1.8 ± 1.3 | 2.2 ± 1.5 | 1.6 ± 1.4 | 0.048 |
| **LVEF (%)** | 56.0 ± 10.1 | 56.8 ± 8.6 | 56.8 ± 10.6 | 56.2 ± 9.9 | 0.84 |
| **LAVi (ml/m^2^)** | 35.9 ± 11.7 | 36.4 ± 11.2 | 42.8 ± 11.3 | 36.5 ± 11.6 | 0.23 |
| **Drugs** |  |  |  |  |  |
| **Class Ic (n, %)** | 87 (16.3%) | 20 (24.7%) | 5 (12.2%) | 112 (19.4%) | 0.53 |
| **Class II (n, %)** | 123 (22.9%) | 24 (29.6%) | 11 (26.8%) | 158 (27.4%) | 0.91 |
| **Class III (n, %)** | 97 (18.1%) | 27 (33.3%) | 17 (41.5%) | 141 (24.5%) | 0.002 |
| **Class IV (n, %)** | 8 (1.5%) | 1 (1.2%) | 2 (4.9%) | 11 (1.9%) | 0.26 |
| **DOAC (n, %)** | 233 (43.5%) | 60 (74.1%) | 24 (58.5%) | 317 (55.0%) | 0.19 |
| **Aspirin (n, %)** | 30 (5.6%) | 5 (6.2%) | 3 (7.3%) | 38 (6.6%) | 0.85 |

AF: atrial fibrillation; BMI: body mass index; CAD: coronary artery disease; DOAC: direct oral anticoagulants; GFR: glomerular filtration rate; LAVi: left atrium volume index; LVEF: left ventricular ejection fraction; TIA: transient ischemic attack. Critical and no critical CAD refers to CT scan.
